# Fabrication of QFN-Packaged Miniaturized GaAs-Based Bandpass Filter with Intertwined Inductors and Dendritic Capacitor

**DOI:** 10.3390/ma13081932

**Published:** 2020-04-20

**Authors:** Jian Chen, Zhi-Ji Wang, Bao-Hua Zhu, Eun-Seong Kim, Nam-Young Kim

**Affiliations:** RFIC Center, Kwangwoon University, 447-1 Wolgye-Dong, Nowon-Ku, Seoul 139-701, Korea; cnjacob@kw.ac.kr (J.C.); erikson37@kw.ac.kr (Z.-J.W.); zhuwangwhy@kw.ac.kr (B.-H.Z.)

**Keywords:** air-bridge structure, bandpass filter, capacitor, gallium arsenide, inductor, integrated passive device, microwave RF, QFN-package

## Abstract

This article presents a compact quad flat no-lead (QFN)-packaged second-order bandpass filter (BPF) with intertwined inductors, a dendritic capacitor, and four air-bridge structures, which was fabricated on a gallium arsenide (GaAs) substrate by integrated passive device (IPD) technology. Air-bridge structures were introduced into an approximate octagonal outer metal track to provide a miniaturized chip size of 0.021 × 0.021 λ_0_ (0.8 × 0.8 mm^2^) for the BPF. The QFN-packaged GaAs-based bandpass filter was used to protect the device from moisture and achieve good thermal and electrical performances. An equivalent circuit was modeled to analyze the BPF. A description of the manufacturing process is presented to elucidate the physical structure of the IPD-based BPF. Measurements were performed on the proposed single band BPF using a center frequency of 2.21 GHz (return loss of 26.45 dB) and a 3-dB fractional bandwidth (FBW) of 71.94% (insertion loss of 0.38 dB). The transmission zero is located at the 6.38 GHz with a restraint of 30.55 dB. The manufactured IPD-based BPF can play an excellent role in various S-band applications, such as a repeater, satellite communication, and radar, owing to its miniaturized chip size and high performance.

## 1. Introduction

With the development of modern wireless communication systems, passive devices, such as balancers, mixers, and power splitters, play an important role in the field of radio frequency (RF)/microwave. Bandpass filters (BPFs) have been vastly studied in the field of microwave filters, as they form an important block of RF/microwave integrated circuits and systems. In addition, the demand for BPF has continued to increase along with an increasing demand for low cost, a wide usage of miniaturization, high performance, and so on [1]. Therefore, researchers have been exploring processing techniques that lead to high precision, mass production, and low cost. 

Recent studies have demonstrated many advanced manufacturing technologies, including low-temperature co-fired ceramics (LTCC), monolithic microwave integrated circuits (MMICs), high-temperature superconductor (HTS), micro-electromechanical systems (MEMS), and micro-fabrication techniques, which promote the development and implementation of BPF design. LTCC is composed of a mixture of various passive components such as hybrid circuits, strip lines, filters, antennas, sensors, transformers, and resonators. LTCC is attractive for BPF fabrication owing to its inexpensive process, and the processing metals have high electrical conductivity and low conductor loss. The disadvantage of LTCC is that the size of the ceramic and the processing plate shrink after firing. In addition, modules must have heat sinks due to the requirement of heat dissipation after heating [2,3]. MMICs based on GaAs and other excellent III-V materials can be used to develop devices operating at the millimeter-wave band, which offers higher functional capabilities, improved system stability, and reduced weight, size, and cost [4]. The main disadvantage of MMIC-based devices is that their performance is inferior compared with that of the device using discrete components [5]. A microstrip BPF based on HTS has a significant advantage in terms of low insertion loss compared with the conventional microstrip BPF owing to its ultra-low surface resistance, leading to a compact and miniaturized design. However, the application of HTS is restricted because of its high cost and complexity as a result of the low-temperature operating environment [6]. MEMSs have become an attractive option for the manufacture of electronic equipment as they can be used for batch production of miniature structures ranging from micrometer to millimeter sizes. A key feature of MEMSs is the ability to faultlessly integrate mechanical and electronic components into one wafer, resulting in reduced cost, size, and weight. Nevertheless, the unreliability of the power-handling capability and ultimate dynamic range significantly shortens the life cycle of MEMS-based devices [7]. 

Integrated passive devices (IPDs) represent a matured technology that has been widely used in the past few years. IPDs offer a good trade-off between systems packaging and modular solutions, providing better overall system performance by integrating other functions. The IPD has the advantages of being a simplified model, having a reduced size, and enhanced performance, benefiting from the reduction of parasitic effects compared with standard discrete systems [8,9]. Moreover, the Q-factor used to evaluate device performances is low for lumped elements but can be enhanced using IPD technology as reported in our previous studies [10,11,12]. GaAs substrates have become the second most important semiconductor materials owing to their high saturated electron drift velocity, low field mobility, low parasitics, and good interdevice isolation. Thus, they can be employed in various systems, including medical treatment, information storage, communications, military application, lasing, and sensing [13,14,15,16,17].

In this study, we designed a quad flat no-lead (QFN)-packaged BPF with an outer intertwined metal track, a center dendritic capacitor, and four air bridge structures, which was fabricated using an IPD processing technique on GaAs substrates. The IPD-based BPF was designed and modeled in the form of an equivalent circuit as presented in Section 2.1, and the fabrication process is described in Section 2.2 to clarify the detailed physical structure of the device. The performance description and comparison of the proposed BPF and the published BPFs are presented in Section 3. QFN-packaging technology was used to protect the device from moisture and ensure good thermal and electrical performances. The packaged device was fixed on the center of a printed circuit board (PCB) using gold wire-bonding for final measurement and evaluation.

## 2. Design and Fabrication 

### 2.1. Design and Circuit Analysis

**Figure 1:**
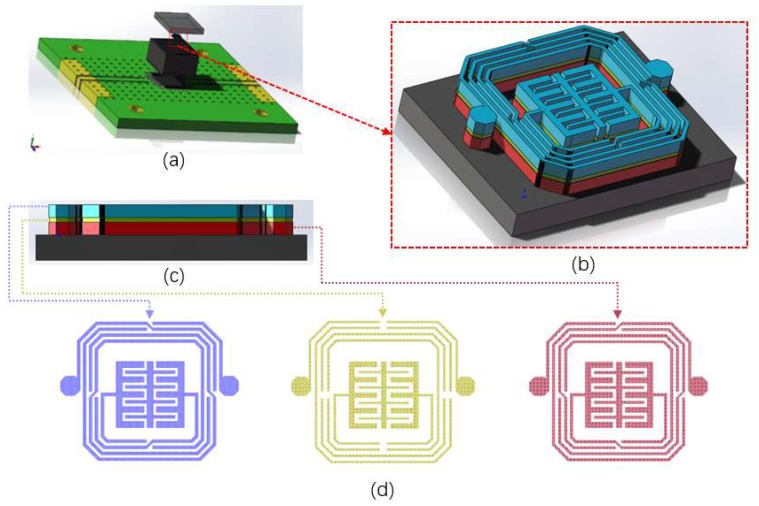
Design of the proposed bandpass filter (BPF): (**a**) 3D view of the packaged BPF on a printed circuit board (PCB) board; (**b**) enlarged view; (**c**) side view; (**d**) three metal layers (leads, text, and bond).

The design, simulation, and verification of the proposed BPF were performed by making full use of advanced design software 2016 (ADS, Keysight Technologies Inc., Santa Rosa, CA, USA). The 3D view of the QFN-packaged BPF mounted on a PCB board is shown in Figure 1a, as well as an enlarged view of the proposed BPF, shown in Figure 1b. A dendritic capacitor was placed in the center of an approximate octagonal outer spiral inductor, and both are composed of three laminated conductor layers. Each metal layer is composed of Cu/Au at a ratio of 9/1. The metal layers are named bond, text, and leads layers from bottom to top. The detailed geometry of the three layers is depicted in Figure 1d. 

In general, the resonant frequency *f_0_* of an LC circuit can be calculated as follows:

(1) $$f_{0}=\frac{1}{2\pi \sqrt{LC}},$$

where *L* is the total inductance and *C* is the total capacitance of the entire device, respectively. After considering the parasitic effect under high-frequency excitation, the lumped model consists of four parts, namely, the non-overlapping part of the metal line, the coupling capacitor between the metals, the induced parasitic effect produced by the SiN_x_ passivation layer and GaAs substrate, and the center capacitor. To accurately evaluate the equivalent circuit, we developed an analytical model for performance evaluation of the proposed BPF using the segmentation method, the mutual inductance method, and the simulated scattering parameters (S-parameters) [18,19]. 

#### 2.1.1. Model inside the Segment Box

**Figure 2:**
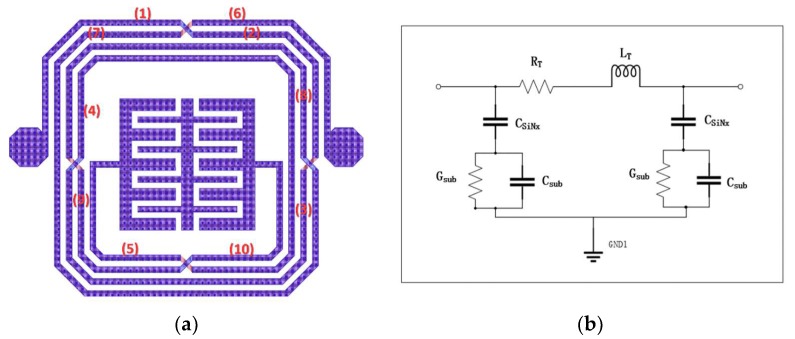

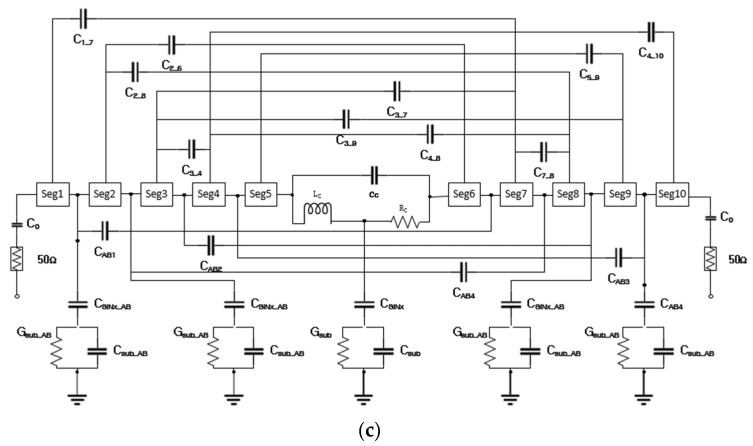
Equipment circuit model of the proposed BPF: (**a**) number of segment boxes of the proposed BPF; (**b**) π-type lumped-element model inside the segment box; (**c**) equivalent circuit model of the proposed BPF.

The intertwined spiral metal track is divided into 10 segments by four air-bridges, as shown in Figure 2a. Then, the equivalent circuit model was established as exhibited in Figure 2b. As shown in the Figure 2c, each segment (Seg i) can be analyzed using the π-type lumped model, where *R_T_* and *Lt* represent the series resistance and series inductance of this segment, respectively, *C_SiNx_* represents the capacitance associated with SiN_x_, and *C_SUB_* and *G_SUB_* represent the capacitance and conductance associated with GaAs, respectively.

The series inductance and series resistance can be determined by the geometry of the metal track and low-frequency resistivity. The current distribution on the track is uneven as the current of the spiral inductor has a superposition effect under high-frequency excitation. Therefore, the inductance and resistance of each segment are related to the instantaneous frequency [20]. Hence, the concept of frequency-dependent effective linewidth *W_eff_* was employed to model the equivalent circuit, which can be calculated as follows:

(2) $$W_{eff}=W_{0,i}\left(1-exp\left(-\frac{w}{W_{0,i}}\right)\right),$$

(3) $$W_{0,i}=c_{1}\times {c_{2}}^{i-1}\sqrt{\frac{1}{f}}\;,$$

where *w* is the physical metal line width, *i* is the number of turns of the coil, and *c*_1_ and *c*_2_ are the fitting parameters to match the resistance and inductance with the measurement results, respectively. The resistance of each metal line (R-line) can be obtained using *W_eff_* as given in Equation (4) to evaluate the signal losses [20],

(4) $$R_{line}=\frac{\rho \times l}{W_{eff}\times t_{m}}\;,$$

where *ρ* is the resistivity (Ω·cm) of the metal, and *tm* and *l* are the thickness and length of the metal line, respectively. The inductance of each segment consists of the self-inductance of the metal line and the mutual inductance with other metal segments. The self-inductance depends on the changing frequency and can be expressed as follows based on *W_eff_* [20]:

(5) $$L_{self}=2l\left(ln\frac{2l}{W_{eff}+t_{m}}-0.5\right).$$

The mutual inductance can be approximated as expressed below, as the metal tracks of each segment are almost parallel to each other [20]:

(6) $$L_{M}=2\times 10^{-4}l\left[ln\left(\frac{l}{d}+\sqrt{1+\frac{l^{2}}{d^{2}}}\right)-\sqrt{1+\frac{l^{2}}{d^{2}}}+\frac{d}{l}\right]\;,$$

where *d* represents the distance between the center positions of every two wires. It should be emphasized that the mutual inductance is positive when the current directions of the two metal tracks are the same, whereas metal tracks with opposite current directions have a negative mutual inductance. Hence, the total inductance associated with length *l* and the desired metal track length can be calculated as given in Equations (4) and (5). 

#### 2.1.2. Models outside the Segment Box

The capacitance effect should be considered, owing to the compact structure of the outer spiral inductor. *C_i_j_* is the coupling capacitance between the adjacent metal tracks, where *i* and *j* are the number of the segments marked in Figure 2a. The capacitor is calculated using the following Equation [21]:

(7) $$C_{i\_j}={\;\varepsilon }_{0}\frac{Q}{V}\;,$$

where *ε_0_* is the vacuum permittivity, and *Q* and *V* are the potentials between the normalized charge and the center position of the two ends, respectively. In addition, the capacitive effect of the four air bridge structures as a result of the air gap between the leads and bond layers cannot be neglected and is expressed as follows [21]:

(8) $$C_{\mathrm{ABi}}=\frac{\varepsilon_{0}\left(\mathrm{overlap}\;\mathrm{area}\right)}{t_{\mathrm{text}}}\;.$$

The two types of capacitors can be evaluated simultaneously using the length calculated in Section 2.1.1 as given in Equations (7) and (8).

#### 2.1.3. Embedded Capacitor

**Figure 3:**
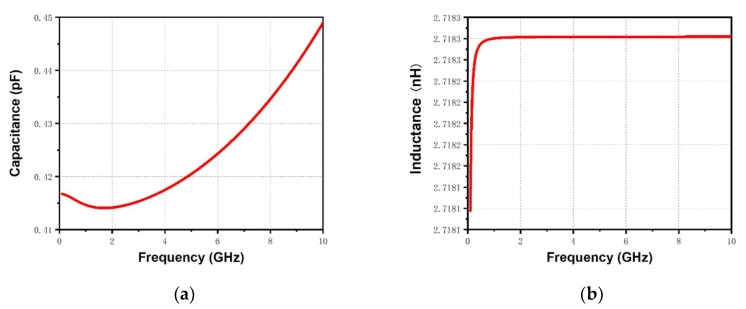

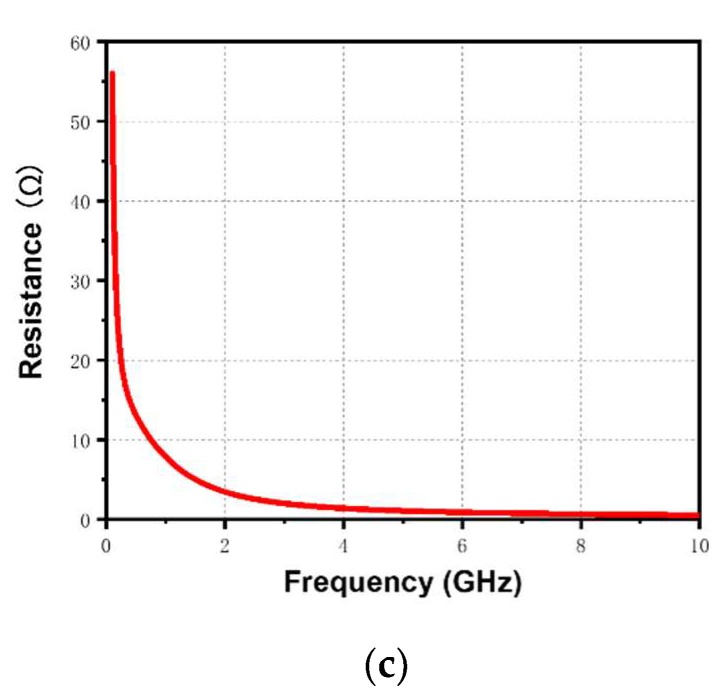
Simulation results of the (**a**) capacitance, (**b**) inductance, and (**c**) resistance of the optimized center capacitor.

The three components of the capacitor *C_C_*, the resistor *R_C_*, and the inductor *L_C_* are introduced to construct the model of the embedded center dendritic capacitor, taking the inductive effect and ohmic loss into consideration. However, the complex structure of the center capacitor results in difficulties in calculating the above parameters. Therefore, the simulated S-parameters and Y-parameters are introduced to calculate the capacitance, resistance, and inductance as given in Equations (9)–(11) [21]. In addition, the capacitance of the center capacitor can be tuned to match the design target by optimizing the size. The frequency-dependent simulation results of the capacitance, resistance, and inductance of the optimized center capacitor are shown in Figure 3.

(9) $$C\left(pF\right)=\frac{1\times 10^{12}\times imag\left[Y\left(1,1\right)\right]}{2\pi f}\;,$$

(10) $$L\left(nH\right)=\frac{1\times 10^{9}}{2\pi f\times imag\left[Y\left(1,1\right)\right]}\;,$$

(11) $$R\left(\Omega \right)=real\left[\frac{1}{Y\left(1,1\right)}\right].$$

#### 2.1.4. Substrate-Related Parasitic Effects

The parasitic effects of the substrate and the passivation layer on the capacitance and inductance should be considered if the design goals are to be achieved. Therefore, three types of the components are introduced in the model, namely, the capacitance associated with the passivation layer (*C_SiNx_* and *C_SiNx_AB_*), the conductance related to the substrate (*G_SUB_* and *G_SUB_AB_*), and the capacitance related to the substrate (*C_SUB_* and *C_SUB_AB_*). The losses caused by eddy currents are negligible since the resistivity of the GaAs substrate is greater than 10 Ω·cm [22]. The above six parameters can be calculated as given in Equations (12)–(17):

(12) $$C_{\mathrm{SiNx}}=\frac{\varepsilon_{0}\varepsilon_{\mathrm{SiNx}}\left(\mathrm{air}\;-\;\mathrm{bridge}\;\mathrm{metal}\;\mathrm{area}\right)}{t_{\mathrm{SiN}x}},$$

(13) $$C_{\mathrm{SUB}\_\mathrm{AB}}=\frac{\varepsilon_{0}\varepsilon_{\mathrm{GaAs}}\left(\mathrm{air}\;-\;\mathrm{bridge}\;\mathrm{metal}\;\mathrm{area}\right)}{t_{\mathrm{GaAs}}},$$

(14) $$G_{\mathrm{SUB}\_\mathrm{AB}}=\frac{\mathrm{air}\;-\;\mathrm{bridge}\;\mathrm{metal}\;\mathrm{area}}{t_{\mathrm{GaAs}}\times \rho_{\mathrm{GaAs}}},$$

(15) $$C_{\mathrm{SiN}x}\left(f\right)=\frac{\varepsilon_{0}\varepsilon_{\mathrm{eff}}\left(f\right)}{2F\left(W_{\mathrm{eff}},{\;t}_{\mathrm{SiN}x}\right)}\times l,$$

(16) $$C_{SUb}\left(f\right)=\frac{\varepsilon_{0}\varepsilon_{eff}\left(f\right)}{2F\left(W_{eff},\;t_{SUB}\right)}\times l,$$

(17) $$C_{\mathrm{SUb}}\left(f\right)=\frac{\sigma_{\mathrm{SUB}}\left(1+\sqrt{1+10t_{\mathrm{SUB}}/W_{\mathrm{eff}}}\right)}{2F\left(W_{\mathrm{eff}},{\;t}_{\mathrm{SUB}}\right)}\times l,$$

where the *SUB__AB_* subscripted components represent the elements related to the air-bridge area, which can be considered as frequency-independent as their lengths are small. In Equations (14)–(16), *ε*_0_, *ε_GaAs_*, and *ε_SiNx_* are the permittivity in free space, of the substrate, and of the passivation layer, respectively, and *t_GaAs_* and *t_SiNx_* are used to indicate the thicknesses of the substrate and passivation layer, respectively. Therefore, a new function *F (W_eff_, t)* is introduced with respect to the effective linewidth, metal thickness, and the frequency-dependent permittivity *ε_eff_(f)* to estimate *C_SiNx_*, *C_SUB_*, and *G_SUB_* [23].

**Figure 4:**
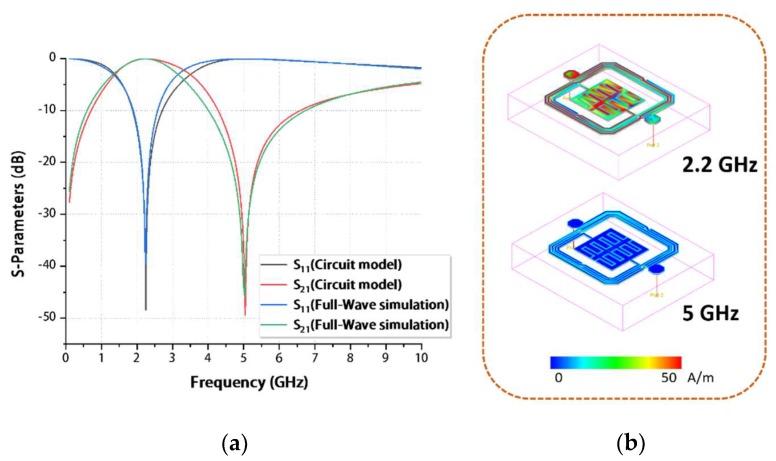
(**a**) S_11_ and S_21_ of the circuit model and full-wave simulations. (**b**) Current density at 2.2 and 5 GHz.

The comparison between full-wave simulation and a proposed circuit model simulation is illustrated in Figure 4a, a good consistency of which validates the circuit analysis mentioned above. In addition, the current densities at 2.2 and 5 GHz are shown in Figure 4b, which are located near the resonance frequency and transmission zero, respectively, further testifying to the accuracy of the full-wave simulation.

### 2.2. Fabrication

**Figure 5:**
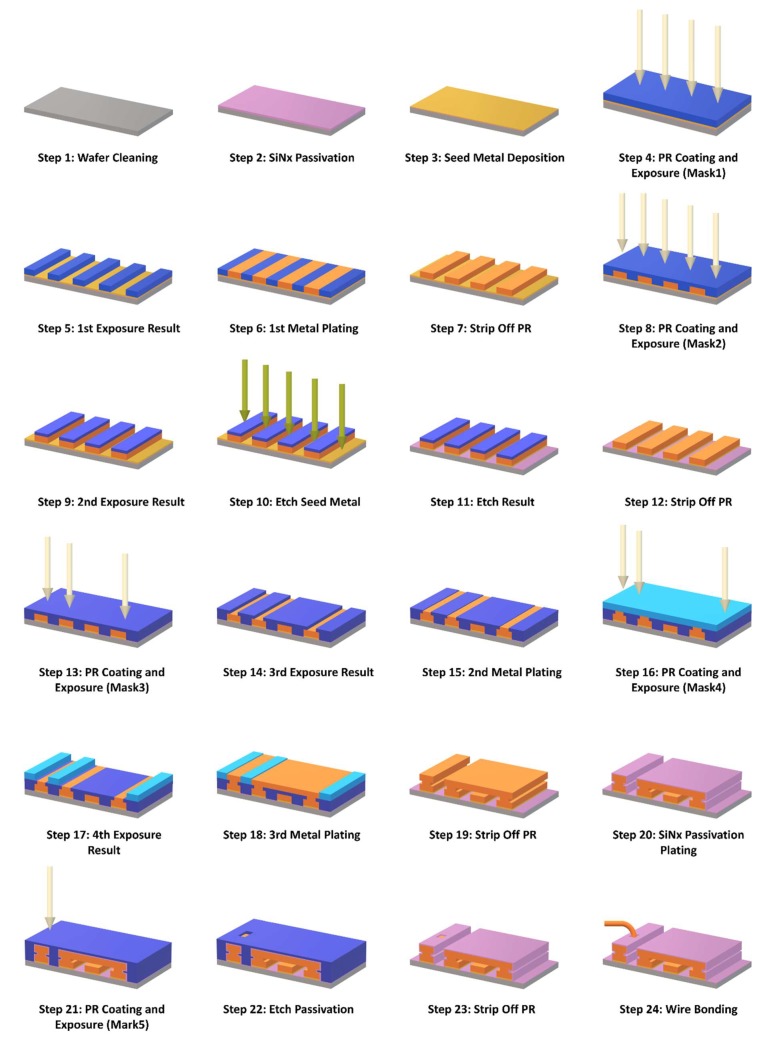
GaAs-based integrated passive device (IPD) technology fabrication flow.

The IPDs were manufactured using standard wafer fabrication techniques combined with thin-film and photolithography processing, which makes the integration of discrete passive electronic components into a chip feasible [24,25]. The BPF proposed in this study was developed using a total of 24 steps and five masks, and the fabrication flow is illustrated in Figure 5. 

First, a 6-in GaAs wafer was cleaned using the lift-off machine with acetone solution to create a flat surface as shown in step 1. Plasma-enhanced chemical vapor deposition (PECVD) technology was used to generate a 200 nm seed metal layer of Ti/Au SiNx passivation layer as shown in step 2; this technique was employed to improve adhesion between the metal layer and the GaAs wafer. Then, a seed metal layer consisting of 20 nm Ti and 80 nm Au was deposited by sputtering, as illustrated in step 3. In step 4, a positive photoresistor (PR) was formed by spin coating and then exposed using mask 1. Then, the first metal layer was formed with Cu/Au using electroplating technology with a thickness of 4.5/0.5 μm, as shown in step 6. Next, the PR was stripped off using acetone solution, as illustrated in step 7. In steps 8–9, the positive PR was subsequently coated again and then exposed using mask 2, which was used to protect the first metal layer from the next etching process. Next, the unused part of the seed metal layer was etched using SF_6_/Ar plasma with an inductively coupled plasma (ICP) etcher, as illustrated in steps 10–12. In step 13, the positive PR was coated again and then exposed using mask 3 to prepare for the second metal layer deposition. Then, the second metal layer of Cu/Au was formed with a thickness of 1.8 μm using electroplating technology, as given in step 15. In step 16, a negative PR, instead of a positive PR, was subsequently coated and then exposed using mask 4 to define the pattern of the third metal layer. Then, in step 17, the third metal layer was electroplated using Cu/Au with a thickness of 4.5/0.5 μm, which is the same as the first metal layer. In step 19, the air-bridge structure was formed, and the PR was removed using acetone solution and wet etching technology. At the same time, other normal three-metal layer structure-like intertwined coils and the center capacitor were also produced. Then, a 300 nm SiN_x_ passivation layer was deposited using PECVD to protect the device from moisture and oxidation, as given in step 20. Subsequently, a positive PR was coated and then exposed using mask 5 to etch the passivation layer via the ICP dry etch process with SF_6_/O_2_ to connect the contacts for further performance testing, as illustrated in steps 21–22. Finally, in step 24, polishing, dicing, and wire-bonding processes were performed to mount the BPF device on the PCB, such that the RF performance of the fabricated device can be measured. The manufacturing techniques used in the IPDs process are explained in detail in Table 1.

## 3. Results and Discussion

**Figure 6:**
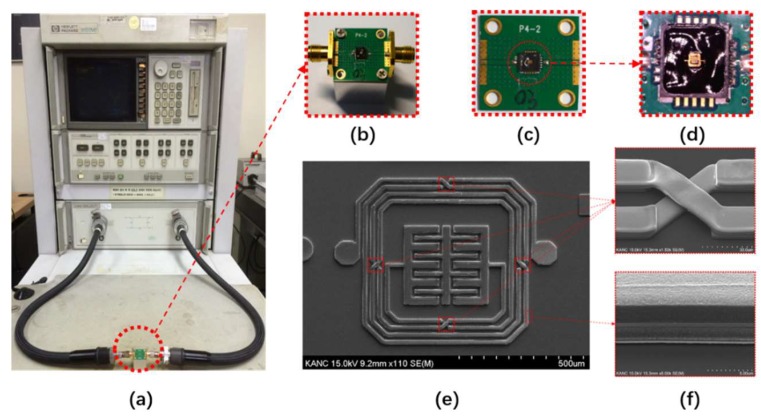
S-parameter measurement setup of the proposed IPD-based BPF with enlarged views of different parts. (**a**) Measurement setup. (**b**) BPF installed on an aluminum cube. (**c**) Top view of the entire PCB. (**d**) Top view of the packaged chip on the PCB. (**e**) Top view of SEM image. (**f**) Enlarged air-bridge area and cross-section of the three metal layers.

The proposed BPF was designed and simulated using the ADS software, fabricated on GaAs substrate with a thickness of 200.1 μm, a dielectric constant of 12.85, and a loss tangent of 0.006, and packaged using the QFN technique. QFN-packaging technology was used to protect the device from moisture and ensure good thermal and electrical performances. S-parameters are used to describe the electrical performance of linear electrical networks when undergoing various steady state stimuli using electrical signals. In particular, it becomes essential to describe a given network in terms of waves at high frequency instead of voltage or current. An Agilent 8510C vector network analyzer (VNA, Keysight Technologies Inc., Santa Rosa, CA, USA) was used to measure and record the transmission and reflection parameters. Figure 6a shows photographs (Nikon D3300 DSLR camera, Nikon, Tokyo, Japan) of the measurement setup; the magnified view of the aluminum test cube (2 × 2 × 2 cm^3^), the complete PCB (2 × 2 cm^2^), and the packaged chip are shown in Figure 6b–d, respectively. The overall dimensions of the pattern are 0.8 × 0.8 mm^2^, and the scanning electron microscope (SEM) images were taken to clearly illustrate the manufactured pattern, as shown in Figure 6e. The enlarged air-bridge area and cross-section of the three metal layers are shown in Figure 6f.

**Figure 7:**
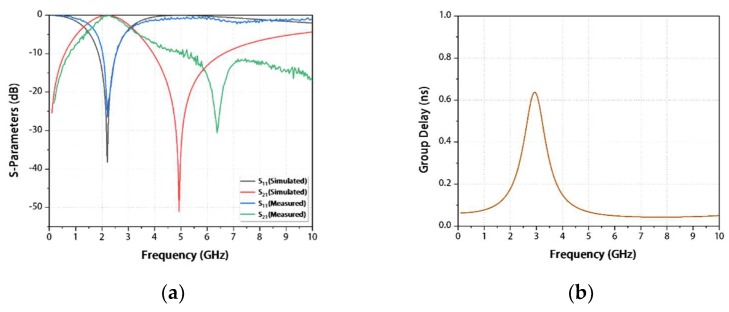
Simulation and measurement results: (**a**) parameters S_11_ and S_21_; (**b**) group delay.

Figure 7a shows the simulated and measured results of the IPD-based BPF. It can be observed that the measured resonant point is located at the frequency of 2.21 GHz, and its 3-dB passband is 1.61–3.20 GHz with a fractional bandwidth (FBW) of 71.94%. The transmission zero is located at the stopband on the right side of the passband, which brings a measured frequency of 6.38 GHz and a magnitude of 30.55 dB. As a considerable figure of merit (FoM) of BPF, the Q-factor is an important indicator to represent the energy lost in the filter, with lower values indicating a smaller loss. The Q-factor is 39.17, which can be derived by Y-Parameters [26,27,28]. The group delay of the entire passband is always smaller than 0.63 ns, as illustrated in Figure 7b [29,30].

Table 2 presents a performance comparison between the proposed BPF and five existing BPFs, indicating that our device has a relatively small chip size, good insertion and return losses, and a relatively wide fractional bandwidth.

Table 3 presents comparisons with other works using various manufacturing technologies, showing that our GaAs-based IPD BPF possesses an ultra-wide passband and smaller dimensions.

## 4. Conclusions

In this study, a microscale QFN-packaged BPF consisting of a centrally embedded capacitor and two intertwined spiral inductors was developed using GaAs-based IPD technology. The equivalent circuit model was established, taking capacitive and inductive parasitic effects into consideration. The three-layer IPD technology fabrication flow using thin-film and photolithography processes was presented in 25 steps. The fabricated BPF highlights a miniaturized overall size of 0.021 λ_0_ × 0.021 λ_0_ (0.8 × 0.8 mm^2^). The measured results show a relatively good consistency with theoretical prediction and simulation, with a low insertion loss of 0.38 dB, and an ultrawide 3-dB FBW of 71.94%. The proposed BPF can be employed in a modern communication system owing to its high performance and miniaturized size as a result of the GaAs-based IPD fabrication technology. However, the lifecycle and compatibility of the proposed BPF in practical application were not investigated in this study; furthermore, its selectivity is limited by it as a low-order device, and its sensitivity will be verified in our future research to promote the practical application of the IPD technology.

## Figures and Tables

**Table 1:** Manufacturing techniques used in the IPDs process.

Fabrication Objective	Technique	Material
Passivation layer	PECVD	SiNx
Photoresistor	Spin-coating	Negative/positive PR
PR removal	Lift-off	Acetone
Seed metal	Sputtering	Ti/Au
Metal layer	Electroplating	Cu/Au
Via	ICP etching	SF_6_/O_6_

**Table 2:** Performance comparison of proposed BPF and published BPFs.

References	Technology	Fractional Band Width (%)	Passband (GHz)	Insertion Loss (dB)	Return Loss (dB)	Circuit Area
[31]	Silicon IPD	107.63 (3 dB)	6.5	1.1	15	0.219 λ_0_ × 0.181 λ_0_
[32]	Silicon IPD	33.33 (3 dB)	2.4	2.3	10	0.024 λ_0_ × 0.024 λ_0_
[33]	Silicon IPD	16 (10 dB)	1.7	2.56	12	0.039 λ_0_ × 0.037 λ_0_
[34]	Glass IPD	49.62 (3 dB)	2.6	0.6	30	0.018 λ_0_ × 0.009 λ_0_
[35]	Glass IPD	36.73 (10 dB)	2.1	3.2	22	0.019 λ_0_ × 0.019 λ_0_
This work	GaAs IPD	71.94 (3 dB)	2.21	0.38	26.45	0.021 λ_0_ × 0.021 λ_0_ (0.8 × 0.8 mm^2^)

**Table 3:** Comparisons between this work and other works using various manufacturing technologies.

References	Technology	Fractional Band Width (%)	Insertion Loss (dB)	Return Loss (dB)	Passband (GHz)	Circuit Area
[36]	Microstrip	13.3	1.1	> 20	0.975	0.094 λ_0_ × 0.08 λ_0_
[37]	HTS	2.6/2.4	0.18/0.32	> 16	1.9/2.6	0.182 λ_0_ × 0.156 λ_0_
[38]	HTCC	5.5	1.8	> 15	2.25	6.9 × 39.9 mm^2^
[39]	LTCC	12.5	2.4	15	2.4	0.058 λ_0_ × 0.058 λ_0_
This work	GaAs IPD	71.94	0.38	26.45	2.21	0.021 λ_0_ × 0.021 λ_0_ (0.8 × 0.8 mm^2^)
